# An active play intervention to improve physical activity and fundamental movement skills in children of low socio-economic status: feasibility cluster randomised controlled trial

**DOI:** 10.1186/s40814-019-0427-4

**Published:** 2019-03-14

**Authors:** Avril Johnstone, Adrienne R. Hughes, Lizann Bonnar, Josie N. Booth, John J. Reilly

**Affiliations:** 1 Physical Activity for Health Group, University of Strathclyde, Glasgow, Scotland G1 1QE UK; 2 School of Psychological Sciences and Health, University of Strathclyde, Glasgow, Scotland G1 1QE UK; 3 Moray House School of Education and Sport, University of Edinburgh, Edinburgh, EH8 8AQ UK

**Keywords:** Active play, Physical activity, Moderate-to-vigorous physical activity, Fundamental movement skills, Inhibition, Academic attainment

## Abstract

**Introduction:**

Active play is a novel approach to addressing low physical activity levels and fundamental movement skills (FMS) in childhood and new interventions must be developed and evaluated.

**Aim:**

This study aimed to determine the feasibility of a 10-week school-based ‘active play’ intervention, and present preliminary findings on four outcomes: physical activity levels, FMS, inhibition, and maths fluency.

**Methods:**

This was a feasibility cluster RCT in which eight schools (one primary three class per school) were paired and randomly allocated to either the 10-week intervention (*n* = 4) or waiting-list control (*n* = 4). The active play intervention consisted of a 1-h outdoor physical activity session per week, incorporating 30 min of facilitated games and 30 min of free play. Feasibility measures were gathered using appropriate methods and physical activity was measured using an ActiGraph GT3X accelerometer, FMS were assessed using the Test of Gross Motor Development-2 (TGMD-2), inhibition was measured using a Flanker Test and maths fluency was assessed using the One Minute Basic Number Facts Test.

**Results:**

Sixty-six percent of eligible children (*n* = 137) agreed to participate in the research. No schools withdrew from the study and three participants were lost to follow-up. Compliance to the intervention was high—none of the participants missed more than two of the ten scheduled active play sessions. Data lost to follow-up were minimal; most were lost (14%) for school day physical activity. Active play sessions were shorter than planned on average by 10 min, and participants spent a mean of 39.4% (14.2) of the session time in moderate-to-vigorous intensity physical activity (MVPA). There was preliminary evidence of a small intervention effect on MVPA (*d* = 0.3), FMS score (*d* = 0.4), inhibition (fish trial: *d* = 0.1, arrow trial *d* = 0.1) and maths fluency (addition: *d* = 0.3, subtraction: *d* = 0.1).

**Conclusion:**

The active play intervention was feasible and benefitted from a relatively high MVPA content; however, preliminary findings suggest the intervention had a small effect on the outcomes. Having more active play sessions per week and/or extending the duration of the intervention may increase the effects and these should be tested before a future definitive cluster RCT is undertaken.

**Trial registration:**

This trial was registered on the International Standardised Randomised Controlled Trials Number register (ISRCTN) in August 2017 (ISRCTN11607781).

## Introduction

It is recommended that UK school-aged children and adolescents (5–18 years) should engage in at least 60 min of moderate-to-vigorous physical activity (MVPA) per day [1]. In Canada, new ‘24-hour movement guidelines’ encourage a whole day approach to movement by recommending that children should engage in four behaviours: ‘sweating, stepping, sleeping and sitting’, at optimal levels to gain the associated health benefits [2]. Specifically, for primary school-age children, a healthy 24 h would include 9–11 h of sleep, 60 min of MVPA, several hours of structured and unstructured physical activity, screen time use of no more than 2 h and a limited amount of time spent sitting [2]. Achieving the UK or Canadian guidelines should bring health benefits, including reducing the risk of some cancers, type 2 diabetes, cardiovascular disease, obesity, mental wellbeing and poor bone health [2–4]. However, children in Scotland and in other high-income countries are typically not achieving the recommended 60 min of MVPA per day [5, 6].

Recent systematic reviews into the contribution of active commuting to school, recess and physical education (PE) on children’s physical activity levels have suggested that they make a small contribution to helping children achieve the physical activity guidelines [7–9]. A recent systematic review found that interventions to promote active play have received little attention in physical activity research to date [10], but the potential of active play for increasing physical activity levels may be substantial given that it can be engaged in before, during and after school, 365 days of the year [11]. Active play is ‘a form of gross motor or total body movement in which young children exert energy in a freely chosen, fun and unstructured manner’ [12]. It is often engaged in outdoors, which is associated with higher habitual physical activity and MVPA levels and is suggested to be one of the factors explaining the higher levels of physical activity in low–middle-income countries compared to high-income countries [6, 13–15]. Furthermore, in high-income countries, those from a lower socio-economic status (SES) typically engage in less active play than those from a higher SES [16, 17].

In addition to increasing physical activity levels, active play also has the potential to improve fundamental movement skills (FMS) [18–20]. FMS are a set of skills, which children should be competent in (such as throwing catching, running and jumping) and competency in these skills is associated with higher physical activity levels [21–23]. Furthermore, research has suggested a possible link between MVPA and improved executive function (i.e. inhibition) and maths attainment [24]. Inhibition is the ability to suppress actions and modify behaviour, which is implicated in many areas of life and learning. Active play has been suggested as a potentially good way of achieving both increased MVPA and improved inhibition [25, 26].

The UK MRC Framework for complex interventions recommends feasibility and pilot research before a definitive randomised controlled trial (RCT) is undertaken [27]. The authors of the present study conducted a pragmatic evaluation of the active play intervention (formally known as Go2Play Active Play) on physical activity and FMS in a non-randomised group of participants [18]. This pragmatic evaluation was sufficiently promising to develop the intervention and evaluation in the form of the present study.

Therefore, the aim of this cluster RCT was to determine the feasibility of an active play intervention to inform a future definitive RCT. Information on consent rate, data lost to follow-up, intervention fidelity and estimates of the effect for each outcome measure (physical activity, fundamental movement skills, inhibition and maths fluency) was collected.

## Methodology

### Trial design

The present study was a two-arm parallel feasibility cluster RCT involving eight primary schools (one primary three class per school) located in Glasgow, Scotland. Glasgow City Council and the funders of the intervention (Inspiring Scotland) chose pupils in primary 3 (aged 7 years) to receive the intervention because this age group receive the least amount of additional physical activity opportunities compared to other age groups. Schools were paired based on relevant criteria and then randomly assigned either to the intervention group or waiting-list control (described in more detail below). Baseline data were collected in August and September 2017 and follow-up data were collected in November and December 2017.

This trial was registered on the International Standardised Randomised Controlled Trials Number register (ISRCTN) in August 2017 (ISRCTN11607781) and follows the CONSORT guidelines for reporting pilot and feasibility trials [28].

Ethical approval was granted by Glasgow City Council’s Education Services and the University of Strathclyde’s School of Psychological Sciences and Health Ethics Committee prior to data collection.

### Procedures

In April 2017, Glasgow City Council invited 32 schools from the South and 28 schools from the North West of Glasgow to participate in the Active Play intervention during the 2017–2018 school year. Thirty-four of the 60 schools agreed to participate in the intervention, and a list of these schools was sent to the lead researcher who divided the schools by location (South and North West). A profile of each school was created by obtaining information held by the Scottish Government (www.gov.scot/Topics/Statistics/Browse/School-Education/Datasets) on socio-economic status (SES) of the school as measured by the Scottish Index of Multiple Deprivation (SIMD) score, percentage of children on free school meals, percentage of children who live in the 20% most deprived areas, school enrolment, number of primary 3 children, percentage of children from ethnic minority groups and if the schools had an existing relationship with the charities delivering the active play intervention.

The aim of the funders, Inspiring Scotland, was to implement the active play intervention in the most deprived schools in Glasgow; for this reason, schools were eligible for this study if at least 70% of pupils from the school were living in the 20% most deprived areas of Scotland. Schools (*n* = 3) were also excluded if they had an existing relationship with any of the charities that delivered the active play intervention (to avoid contamination) or had been involved in the previous pragmatic evaluation [18]. Once the schools not meeting these criteria were excluded, five schools remained from the South of the city and six schools from the North West, at which point they were paired on deprivation (based on the percentage of children who live in the 20% most deprived areas), school enrolment, demographics (percentage of children from ethnic minorities) and geography (located close to each other). Two schools in the North West and one school in the South of the city were removed and kept for contingencies because they were not located near the other schools. Therefore, eight schools were selected for the study and all of these eight schools agreed to take part in the study via their head teachers.

Each pair of schools were randomised prior to data collection beginning. A researcher unaffiliated to the present study used a random number generator to randomly assign each pair to either the intervention or the waiting-list control. Schools allocated to the intervention group were informed they would receive the intervention starting in August 2017 and the control schools would receive the intervention in April 2018 once the research was completed. Eight schools were involved in the study due to limitations on time and resources; this was considered sufficient for a feasibility trial as previous studies have also used a similar number of clusters [29].

Information and consent forms were distributed to all children in the primary 3 class of each school. Children were asked to pass on the consents to their primary care giver, who then provided consent (by signing the consent form and returning it to the teacher in the child’s school bag) if they wished their child to participate in the research. The class teacher then gathered the consents for the researcher who collected them in early August 2017. Children orally confirmed that they would like to participate in the research and were reminded that they could opt out of the research without affecting their participation in the active play intervention. All children participated in the active play intervention, but only consenting children participated in the study. Participants were eligible for the study if they were apparently healthy and able to participate in active play unaided. Participants’ weight status, SES using the SIMD [30], FMS, inhibition and maths fluency measured 2 weeks before the intervention began, and physical activity was measured 1 week before the intervention began. At week 9 of the intervention, physical activity was measured again, and the other outcome measures were assessed once the intervention was completed.

### Intervention

Inspiring Scotland (www.inspiringscotland.org.uk, Edinburgh) and Agile CIC (www.agilecic.com, Glasgow) developed the active play (formally known as Go2Play Active Play) intervention in 2014. In the present study, the intervention was delivered by playworkers from two local play charities who were trained by Agile CIC. The intervention has been detailed previously [18], but briefly, it is underpinned by the concept of physical literacy. Physical literacy is ‘the motivation, confidence, physical competence, knowledge and understanding to value and take responsibility for engagement in physical activities for life’ [31]*.* Key to establishing a foundation of good physical literacy is developing children’s physical competency (i.e. FMS) and ensuring they have a positive experience of physical activity from an early age [32–34]. Increasing levels of MVPA and improving fundamental movement skills are the main aims of the present intervention.

The active play intervention was delivered to one primary 3 class per school for 10 weeks (one session per week). The intervention was planned to consist of a 1-h outdoor physical activity session: 30 min of facilitated games plus 30 min of free play. The play charities were supplied with a standard set of basic equipment, which included a range of balls, tennis racquets, hockey sticks, skipping ropes among other items to enable them to deliver both elements of the intervention. This equipment was not left with schools to use between sessions. During the facilitated section of the session, the playworkers led and joined in on games designed to develop participants’ FMS and other components of physical literacy (examples of games can be found at www.actify.org.uk/activeplay). During the free play section of the session, the equipment was provided, and participants were free to choose what they wanted to play. The playworkers and teachers were encouraged to participate fully in the sessions with the children. The delivery principles of the active play sessions are that they should be fun, inclusive and active, which should encourage high levels of MVPA and FMS development [18].

Although children only received one session per week, the intervention might increase physical activity levels beyond the session as the equipment is basic, inexpensive and readily available at home or school; children are learning to play which may encourage play outside intervention time; and improving FMS and other aspects of physical literacy might facilitate increased physical activity [22, 23].

### Outcomes

#### Anthropometrics

All consenting participants had their height and weight measured at baseline only (to the nearest 0.1 cm/kg) using a portable stadiometer and digital scales (both Seca, Hamburg, Germany). Weight status is presented as a BMI z-score relative to 1990 UK reference data; healthy weight (BMI z-score < 1.04); overweight (BMI z-score 1.04–1.64); and obese (BMI z-score > 1.64).

#### Feasibility measures

The total number of children in each class was provided by the class teacher. From this, the percentage of children who consented from the total sample available was calculated by the lead researcher.

Feasibility of the outcome measures was also captured by the lead researcher who kept a record of the number of children who provided data at baseline for each outcome measure, and the number, with explanations, that were lost to follow-up (for example, moved school, no longer wanted to participate in research, data not valid).

To determine if the intervention was delivered as intended, the playworkers kept a record of the number of sessions they delivered, if any sessions were delivered indoors due to adverse weather conditions, how long the sessions lasted and if any child was injured because of participating in active play. Additionally, the lead researcher observed the playworkers delivering one session per school at week 4 or 6 of the intervention to determine if they were delivered as intended. To support observations, an assessment tool was developed by Agile CIC, which assessed four key dimensions to delivering a successful session: team/individual skills and attributes (for example, demonstrates confidence and enthusiasm), knowledge and experience (for example, demonstrates experience in leading play and physical activity sessions), putting the training into practice (for example, plans and delivers appropriate session for age group) and delivery (for example, session incorporates a range of FMS and are fun, inclusive and active). For each of these four dimensions, there were 4–6 items in which the playworkers were scored out of 5. Class teachers were asked to record attendance at the active play sessions to determine how many sessions each participant attended.

#### School day physical activity

Physical activity was measured using an ActiGraph GT3X accelerometer (Pensacola, Florida, USA), which is a small and unobtrusive monitor attached to an elastic waist-belt worn over the participant’s right hip [35–37]. Data were collected in 15-s epochs and raw physical activity data were converted to total volume of physical activity (counts per minute—cpm) and time (minutes/school day) spent in sedentary (0–100 cpm), light (101–2292 cpm) and MVPA (≥ 2293 cpm) intensities using Evenson cut points [35]. Evenson cut points have evidence of validity and reliability for children and adolescents across varying physical activity intensities [35, 38].

Participants were asked to wear the ActiGraph accelerometer for five school days (9.00–15.00), except for one pair of schools who wore the monitors for four school days. Teachers helped the children attach the accelerometers. Each child was assigned a specific monitor (denoted by a number displayed on the monitor) and a list of each child and their specific monitor was noted in a handbook supplied to each class teacher involved in the research to ensure each child wore the same device each day. Class teachers reported the time the monitors were attached and removed each day in their handbook, and these times were used to extract the raw data from the monitors. Participants had to wear the monitors for a minimum of four school hours and for at least 3 days for the data to be valid, the same criteria used in our previous study and in other school-based studies [18, 39]. The average actual wear time during school-time was 5.4 h per day and 4.5 days at baseline and 5.3 h per day and 4.3 days at follow-up.

Each pair of schools had their physical activity measured during the same week at baseline and follow-up and were measured the week prior to the intervention beginning at baseline and during week 9 of the intervention for follow-up. However, one intervention school was measured at week 8 of the intervention as their first session was cancelled and two control schools had to be measured again in January 2018, as they did not wear the monitors during week 9 (i.e. November and December 2017) as planned.

#### Physical activity content of active play

During the follow-up physical activity data collection week which took place on week 8 (*n* = 1 school) or 9 (*n* = 3 schools) of the intervention, the lead researcher attended the intervention sessions to note the time (to the nearest minute) the session started, finished and when the facilitated games part finished, and free play began. These times were then used to accurately extract accelerometer data to determine the physical activity content of the sessions in terms of percent time spent in sedentary behavior, light intensity physical activity and MVPA.

#### Fundamental movement skills

FMS were assessed using the Test of Gross Motor Development-2 (TGMD-2), which is divided into two subtests: locomotor (run, gallop, hop, leap, horizontal jump, sidestep) and object control (strike, dribble, catch, kick, throw, underhand roll) [40]. Each skill is comprised of 3–5 components based on a model performance of how the skill should be performed. If the participant performed each component as described, they were scored a ‘1’, or a ‘0’ if they did not [40].

FMS were assessed, predominately outdoors, by the same lead researcher prior to the intervention beginning and after the intervention had finished. During the assessment, the lead researcher demonstrated the skill once and then participants performed each skill twice while being observed and scored accordingly [40]. Scores were then adjusted for age and gender to give standard scores and percentiles for the locomotor and object control skills, which are then totalled to give a gross motor quotient score (GMQ—total FMS score) and a percentile [40].

In instances where more than 20 children consented from a school (*n* = 1 school), 10 male and 10 female participants were randomly selected using a random number generator to have their FMS assessed due to time restrictions.

#### Inhibition

Inhibition was measured using the NIH Toolbox Flanker Test, which was administered on an Apple iPad Air 2 (Apple Inc., California, USA). The Flanker Test consisted of a mix of congruent (all stimuli facing in the same direction) and incongruent trials (the middle stimulus is facing in the opposite direction to the flanker stimuli) and participants were asked to select the button on the screen that matched the direction of the middle stimulus [41]. Participants were given four practice trials and if they got > 1 one trial incorrect, they received a further four practice trials. The test consisted of 20 trials where the stimuli were fish and if they scored 18/20 correct, they then completed another test involving 20 trials where arrows were the stimuli (12 congruent and 8 incongruent trials for both tests) [41]. Participants were given a maximum of 10 s to respond in each trial; if they did not respond within this timeframe, then the screen moved on to the next trial.

Practice and non-response trials were removed, and accuracy scores were calculated for the fish test and the arrow test separately (average accuracy score). Trials with incorrect responses were then removed and reaction time (s) was averaged for the fish test and the arrow test separately for the congruent and incongruent trials. Finally, the mean reaction time for the congruent trials was subtracted from the mean reaction time for the incongruent trials for the fish test and the arrow test separately to calculate the conflict score (i.e. the measure of inhibition) for the fish test and the arrow test for each participant.

#### Maths fluency

Maths fluency was measured using the One Minute Basic Number Facts Test (1995), which was a simple pencil and paper test that assessed participants’ addition and subtraction abilities [42]. Participants were asked to answer as many addition sums as possible in 1 min by writing their answers next to sums [42]. The same protocol was then followed for the subtraction element of the test [42]. The number of correct answers was then totalled separately for the addition and subtraction component of the test [42]. Normative values recommend that a child aged 7 years old should be scoring 8 points for addition and 6.5 points for subtraction.

Inhibition and maths fluency were measured 2 weeks prior to the intervention beginning and again at follow-up once the intervention was complete. Participants were assessed in small groups in a quiet room supplied by each school and the order of assessments conducted was randomised to minimise order effects; for example, if one group completed the inhibition measure first, the following group completed the maths fluency first. The same protocol was followed in most instances at follow-up; however, at the start of baseline data collection, there was a delay in obtaining the NIH Toolbox Flanker Test, which resulted in the researchers arranging to go back and measure schools (*n* = 3) on a separate day. We did not measure inhibition on a separate day for follow-up in these three schools.

### Blinding

Standardised procedures were followed for each outcome measured at baseline and follow-up. The lead researcher was not blinded to any of the outcome measures; however, the lead researcher could not influence the physical activity, inhibition and maths fluency measures. For the FMS outcome, the same researcher assessed each participant at both baseline and follow-up following standardised procedures.

### Data analysis

Data analyses were completed using SPSS v 23.0 (SPSS Inc., Chicago, IL). Initial tests for normality were conducted to determine if data were normally distributed (skewness and kurtosis < |2.0|). To explore the potential effect of the intervention, preliminary analyses were conducted to obtain the mean change and 95% CIs for the change in each outcome from baseline to follow-up within each group and between groups (i.e. the mean difference between the groups for the change and the 95% CI) and effect sizes were calculated using Cohen’s *d*; *d* > 0.2 is a small effect size, *d* > 0.5 is medium and *d* > 0.8 is a large effect size. Since inhibition was not normally distributed, the median change from baseline to follow-up within each group and between groups (i.e. the mean difference between the groups for the change and the 95% CI) was obtained and effect sizes were calculated using Cohen’s *d*. The statistical analysis was completed by the lead author and supported by an experienced statistician.

## Results

### Baseline characteristics

**Fig. 1:**
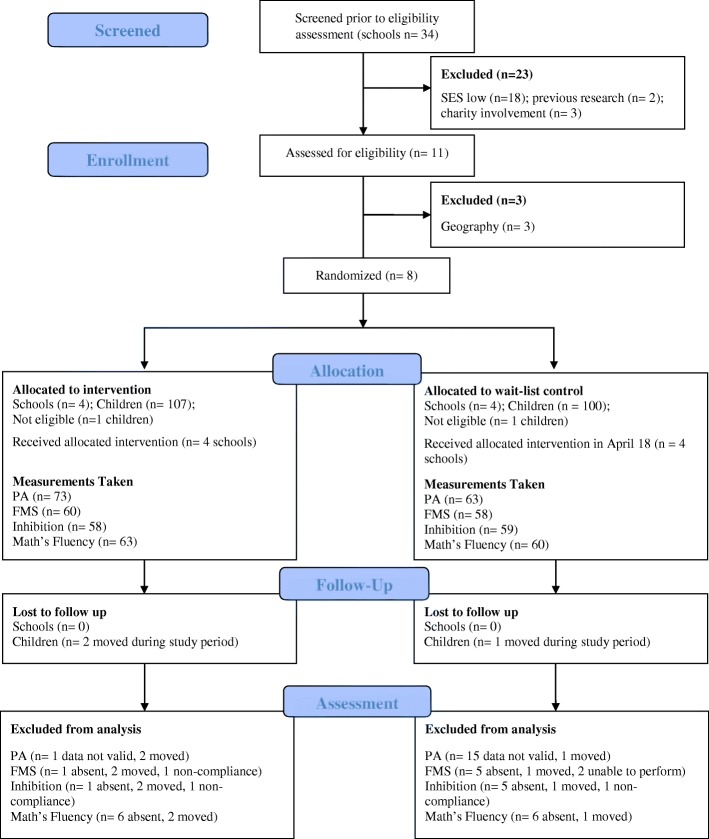
CONSORT diagram showing the flow of schools and participants through each stage of the cluster RCT

Figure 1 shows the flow of schools and participants through the study. As Fig. 1 highlights, a total of 207 children from the primary 3 class of each school were invited to take part in the research (2 participants were not eligible as they had a disability which may have affected their ability to engage in active play). A total of 137 children (intervention *n* = 73; control *n* = 64) consented to participate in the study, a consent rate of 66%. The consent rate varied across schools and was marginally higher in the intervention group (68%; school 1 = 65%, school 2 = 73%, school 3 = 90%, school 4 = 43%) compared to the control (64%; school 5 = 61%, school 6 = 73%, school 7 = 59%, school 8 = 63%). Baseline characteristics of the consenting participants were similar in the intervention and control group as presented in Table 1.

**Table 1:** Demographics of consenting participants

	Intervention (*n* = 73)	Control (*n* = 64)
	Mean (SD) or *n* (%)	Mean (SD) or *n* (%)
Male	34 (47%)	24 (38%)
Female	39 (53%)	40 (62%)
Age (years)	7.1 (0.3)	7.0 (0.3)
BMI z-score	0.7 (1.2)	0.5 (1.3)
*n*(%) living in top 20% most socio-economically deprived areas of Scotland	49 (72%) 5 missing	51 (85%) 4 missing

### Feasibility outcomes

Figure 1 shows that no schools were lost to follow-up and three children moved from their schools during the study period (*n* = 2 from the intervention group and *n* = 1 from the control group). The number of participants providing data for each outcome at baseline and lost to follow-up is also presented in Fig. 1. Data lost to follow-up were minimal in most instances, and when data were lost to follow-up, this was predominantly due to pupil absences on measurement days for each of the outcomes. Most data were lost (14%; *n* = 3 for the intervention group and *n* = 16 for the control group) for the school day physical activity outcome, which was largely due to participants not wearing the monitor for the minimum wear time as specified in the Methods section. Physical activity was measured at baseline during August and September and follow-up during November and December; however, physical activity had to be re-measured in two control schools during January 2018, as they did not wear the monitors during week 9 as planned.

All schools received 10 weeks of the intervention; however, one school had a session cancelled during week 1, which meant (as previously stated) physical activity measurements were taken at week 8 and this school received two sessions during the final week. One school purchased the services of one play charity to provide more active play opportunities throughout their school during the research period, which involved a combination of recess games and play leadership on 1 day of the week. The primary 3 class from this control school did not receive any play leadership but might have engaged in activities during recess on the day the play charity provided activities.

Attendance at the active play sessions was high, only four participants missed two sessions and no participants missed more than two sessions. All sessions took place outdoors but tended to be shorter than the 1 h, by 10 min on average, due to class teachers bringing the participants to the sessions late because they had to walk from their class to where the session was being delivered in the playground.

Playworkers from both play charities scored highly in the assessment tool, with play charity A scoring 3.6 and play charity B scoring 4.7 out of 5. The main area where playworkers needed to improve was in the delivery aspect of the assessment tool. The sessions would have been further enhanced if the playworkers increased their confidence through expanding their knowledge of the intervention and greater practice of facilitating the sessions. See Table 2 for each charity’s score on the assessment tool.

**Table 2:** Assessing capacity of play charities to deliver active play

	Charity A	Charity B
Team/individual skills and attributes	3.8	5.0
Knowledge and experience	3.6	4.6
Putting the training into practice	3.7	4.8
Delivery	3.2	4.4
Total	3.6	4.7

Scores out of 5; see appendix one for a copy of the blank assessment

### School day physical activity

Table 3 presents the results of the preliminary analyses of the between- and within-group effects of the intervention.

**Table 3:** Percent of school day spent in sedentary behaviour, light intensity physical activity, and MVPA at baseline and follow-up in the intervention and control group

	Intervention (*n* = 70)			Control (*n* = 48)			Difference between groups for the change	
	Baseline Mean (SD)	Follow-up Mean (SD)	Within group change Mean; *p* value; *d*	Baseline Mean (SD)	Follow-up Mean (SD)	Within group change Mean; *d*	Mean (95% CI)	*d*
Sedentary %	51.2 (8.6)	49.1 (8.9)	− 2.1; 0.3	55.1 (8.9)	52.4 (9.0)	− 2.7; 0.4	0.6 (− 1.8, 3.1)	0.1
Light %	40.0 (6.5)	40.7 (6.7)	+ 0.7; 0.2	38.5 (7.2)	40.6 (7.8)	+ 2.1; 0.4	− 1.4 (− 3.3, 0.6)	0.3
MVPA %	8.8 (3.4)	10.2 (3.9)	+ 1.4; 0.5	6.4 (3.3)	7.0 (3.0)	+ 0.6; 0.3	0.8 (− 0.2, 1.8)	0.3

There was preliminary evidence of a small intervention effect on percent school time in sedentary behavior (+ 0.6; 95% CI − 1.8, 3.1; *d* = 0.1), light intensity physical activity (− 1.4; 95% CI − 3.3, 0.6; *d* = 0.3) and MVPA (+ 0.8; 95% CI − 0.2, 1.8; *d* = 0.3) in the intervention group compared to the control group.

There was a decrease in percent time in sedentary behaviour (− 2.1%; 95% CI − 3.7, − 0.6;*d* = 0.3) and an increase in light intensity physical activity (+ 0.7; 95% CI − 0.5, 2.0; *d* = 0.2) and MVPA (+ 1.4%; 95% CI: 0.8, 2.0; *d* = 0.5) in the intervention group. The control group also had a decrease in percent time in sedentary behaviour (− 2.7%; 95% CI − 4.6, − 0.8;*d* = 0.4), and an increase in light intensity physical activity (+ 2.1%; 95% CI 0.6, 3.6; *d* = 0.4) and MVPA (+ 0.6; 95% CI − 0.1, 1.4; *d* = 0.3).

### Physical activity content of the active play intervention

Means and standard deviations for the percent time spent in sedentary, light and MVPA during an active play session measured at week 8 or 9 of the 10-week intervention are presented in Table 4.

**Table 4:** Percent time spent in sedentary behaviour, light intensity physical activity and MVPA during the active play session

Intervention (*n* = 68)	Full session Mean (SD)	Facilitated games Mean (SD)	Free play Mean (SD)
Sedentary %	13.2 (7.8)	16.9 (8.0)	10.5 (9.7)
Light %	47.4 (10.6)	45.6 (9.6)	48.1 (16.1)
MVPA %	39.4 (14.2)	37.6 (12.3)	41.3 (20.8)

Participants spent an average of 39.4% (14.2) of their time in MVPA during the full session, and 37.6% (12.3) and 41.3% (20.8) of their time in MVPA during the facilitated games and free play component, respectively.

### Fundamental movement skills

Table 5 presents the results of the preliminary analyses of the between- and within-group effects of the intervention.

**Table 5:** Fundamental movement skills scores at baseline and follow-up in the intervention and control group

	Intervention (*n* = 56)			Control (*n* = 50)			Difference between groups for the change	
	Baseline Mean (SD)	Follow-up Mean (SD)	Within group change Mean; *d*	Baseline Mean (SD)	Follow-up Mean (SD)	Within group change Mean; *d*	Mean (95% CI)	*d*
GMQ score	87.7 (12.8)	90.8 (10.5)	3.1; 0.4	92.0 (12.1)	92.0 (10.1)	0.0; 0.0	3.2 (− 0.1, 6.4)	0.4
GMQ percentile	26.5 (23.6)	30.8 (20.1)	4.3; 0.3	34.3 (23.5)	33.2 (19.9)	− 1.1; 0.1	5.4 (− 1.3, 12.0)	0.3
Locomotor score	8.6 (2.5)	9.0 (1.9)	0.4; 0.2	9.2 (2.5)	8.7 (1.8)	− 0.4; 0.2	0.8 (0.1, 1.6)	0.4
Locomotor percentile	34.7 (25.3)	38.5 (21.0)	3.8; 0.2	40.7 (26.1)	35.5 (19.7)	− 5.2; 0.2	8.9 (0.9, 17.0)	0.4
Object control score	7.3 (2.6)	7.9 (2.3)	0.6; 0.4	8.2 (2.3)	8.6 (2.0)	0.4; 0.2	0.2 (− 0.4, 0.9)	0.1
Object control percentile	24.4 (20.9)	29.5 (21.4)	5.1; 0.3	32.0 (22.1)	35.5 (19.8)	3.5; 0.2	1.6 (− 4.8, 8.0)	0.1

### GMQ

There was preliminary evidence of a small intervention effect on GMQ score (+ 3.2; 95% CI − 0.1, 6.4; *d* = 0.4) and percentile (+ 5.4; 95% CI − 1.3, 12.0; *d* = 0.3).

The intervention group had an increase in GMQ score (+ 3.1; 95% CI 0.9, 5.3; *d* = 0.4) and percentile (+ 4.3; 95% CI − 0.3, 8.8; *d* = 0.3). There was no increase in GMQ score (0.0; 95% CI − 2.4, 2.3; *d* = 0.0) or percentile (− 1.1; 95% CI − 5.9, 3.7; *d* = 0.1) in the control group.

### Locomotor and object control skills

There was preliminary evidence of a small effect on locomotor score (+ 0.8; 95% CI 0.1, 1.6; *d* = 0.4) and percentile (+ 8.9; 95% CI 0.9, 17.0; *d* = 0.4). There was also a limited effect on object control score (+ 0.2; 95% CI − 0.4, 0.9; *d* = 0.1) and percentile (+ 1.6; 95% CI − 4.8, 8.0; *d* = 0.1).

The intervention group had an increase in locomotor skill score (+ 0.4; 95% CI − 0.1, 0.9; *d* = 0.2), locomotor percentile (+ 3.8; 95% CI − 1.8, 9.3; *d* = 0.2), object control score (+ 0.6; 95% CI 0.2, 1.1; *d* = 0.4) and object control percentile (+ 5.1; 95% CI 0.7, 9.5; *d* = 0.3). The control group did not have an increase in locomotor skill score (− 0.4; 95% CI − 1.0, 0.1; *d* = 0.2) or locomotor percentile (− 5.2; 95% CI − 11.0, 0.7; *d* = 0.2), but their object control score (+ 0.4; 95% CI − 0.1, 0.9; *d* = 0.2) and object control percentile (+ 3.5; 95% CI − 1.1, 8.2; *d* = 0.2) increased.

### Inhibition

Table 6 presents the results of the preliminary analysis of the between- and within-group effects of the intervention.

**Table 6:** Inhibition scores at baseline and follow-up in the intervention and control group

	Intervention (fish *n* = 54; arrow *n* = 51)			Control (fish *n* = 52; arrow *n* = 45)			Difference between groups for the change
	Baseline Median (IQR)	Follow-up Median (IQR)	Within group change Median; *d*	Baseline Median (IQR)	Follow-up Median (IQR)	Within group change Median; *p*-value; *d*	*d*
Fish accuracy	100% (100, 100%)	100% (96, 100%)	0%; 0.0	100% (94, 100%)	100% (100, 100%)	0%; 0.4	0.4
Arrow accuracy	100% (94, 100%)	100% (100, 100%)	0%; 0.4	100% (96, 100%)	100% (100, 100%)	0%; 0.1	0.3
Fish trials conflict score	0.13 (− 0.01, 0.28)	0.12 (0.05, 0.2)	− 0.05; 0.1	0.17 (0.08, 0.32)	0.08 (0.01, 0.21)	− 0.07; 0.3	0.1
Arrow trials conflict score	0.24 (0.11, 0.46)	0.15 (0.10, 0.30)	− 0.08; 0.3	0.38 (0.10, 0.74)	0.21 (0.01, 0.46)	− 0.18; 0.3	0.1

There was preliminary evidence of a small intervention effect on the change in accuracy score for the fish trials (*d* = 0.4) and arrow trials (*d* = 0.3).

There was a ceiling effect in the intervention group for accuracy score for the fish trials (*d* = 0.0) and the arrow trials (*d* = 0.4). The control group also had ceiling effects for the accuracy score for the fish trials (*d* = 0.4) and the arrow trials (*d* = 0.1).

There was preliminary evidence of a limited intervention effect on the change in conflict score for the fish trials (*d* = 0.1) and the arrow trials (*d* = 0.1).

The intervention group had a small improvement in conflict score for the fish trials (*d* = 0.1) and the arrow trials (*d* = 0.3). The control group also improved their conflict score for the fish trials (*d* = 0.3) and arrow trials (*d* = 0.3).

### Maths fluency

Table 7 presents the results of the preliminary analysis of between- and within-group effects of the intervention.

**Table 7:** Maths fluency: addition and subtraction scores at baseline and follow-up in the intervention and control group

	Intervention (*n* = 57)			Control (*n* = 53)			Difference between groups for the change	
	Baseline Mean (SD)	Follow-up Mean (SD)	Within group change Mean; *d*	Baseline Mean (SD)	Follow-up Mean (SD)	Within group change Mean; *d*	Mean (95% CI)	*d*
Addition	8.8 (5.0)	12.4 (5.9)	3.6; 1.0	8.2 (4.4)	10.8 (5.5)	2.6; 0.8	1.0 (− 0.3, 2.3)	0.3
Subtraction	5.9 (4.4)	9.3 (4.3)	3.4; 1.2	4.2 (3.3)	7.3 (4.3)	3.1; 0.9	0.3 (− 0.9, 1.5)	0.1

There was preliminary evidence of a small intervention effect on the change for addition scores (+ 1.0; 95% CI − 0.3, 2.3; *d* = 0.3) and subtraction scores (+ 0.3; 95% CI − 0.9, 1.5; *d* = 0.1).

The intervention group had an increase in addition scores (+ 3.6; 95% CI 2.7, 4.5; *d* = 1.0) and subtraction scores (+ 3.4; 95% CI 2.6, 4.3; *d* = 1.2). The control group also had an increase in addition scores (+ 2.6; 95% CI 1.6, 3.5; *d* = 0.8) and subtraction scores (+ 3.1; 95% CI 2.3, 4.0; *d* = 0.9).

## Discussion

The present study was a feasibility cluster RCT designed to inform a future definitive cluster RCT; therefore, the sample size was not designed to detect intervention effects. Information collected on the feasibility outcomes suggested that the present study is feasible. The study benefitted from a relatively high pupil consent rate of 66%, a 100% school retention rate and the loss of only three pupils (as they moved school) at follow-up. Compliance to the intervention was high, only four participants from the intervention group missed two sessions and none missed more than two. Compliance to the outcome measures was also high; 14% (*n* = 3 for the intervention group and *n* = 16 for the control group) of data were lost for the school day physical activity outcome, which was predominantly due to participants not wearing the monitor for the minimum wear time as specified in the ‘Methods’ section. Furthermore, two control schools had physical activity re-measured in January 2018. The playworkers who implemented the intervention scored highly in the assessment tool, but confidence could have been higher in the delivery of sessions. Furthermore, the sessions were often shorter than intended by approximately 10-min per session, which equates to a total of 1-h and 40 min over the 10 weeks. Sessions were shorter because teachers brought the children late to the sessions, particularly when an active play session followed afternoon recess. The low levels of participants lost to follow-up, data lost to follow-up and high compliance to the intervention might be partly explained by the benefits of delivering the intervention and collecting outcome data in a school setting.

The present study had a consent rate of 66% which was reasonable in comparison to other similar studies; a non-school-based active play intervention conducted by O’Dwyer et al. [43] had a consent rate of 42%. Participant dropout rate was comparatively lower in the present study than other studies, with only three participants dropping out as they moved school during the course of the study [44]. Data lost at follow-up varied across the outcomes, but most was lost (14%) for the physical activity outcome, which was marginally higher than similar studies [19, 44]. In a future definitive trial, the timings of the measurements should be considered as two schools originally scheduled to have their follow-up physical activity had to be re-measured in January. This is because December is often a busy period for schools, and therefore it might not be suitable to measure physical activity during this month in Scottish schools. Participants appeared to be receptive to the active play intervention with only four participants missing two sessions and none missed more than two. In summary, the procedures for conducting the present feasibility RCT appear to be mostly suitable for a future definitive trial.

The active play intervention benefits from a collaborative approach in which local play charities deliver the sessions to enable teachers to participate, learn and then embed the intervention beyond the 10 weeks. Furthermore, utilising charities who are experts in play might increase the likelihood that children will continue playing at home and in their communities, particularly as the equipment provided is likely to be similar to what children might have access to at home.

The active play intervention was promising in terms of the MVPA content, with participants spending on average 39.4% of the session in MVPA. Interestingly, the participants engaged in slightly more MVPA during the free play component of the session compared to the facilitated games component (41.3% of time in MVPA on average versus 37.6%). The previous study conducted by Johnstone et al. [18] found that the participants spent 30.1% of their time during an active play session in MVPA and Brazendale et al. found that during a 1-h session of solely free play, participants spent 25% of that time in MVPA [45]. To put this in context, a recent systematic review suggested that during physical education, participants of primary school-age children typically spend as little as 11% of their time in MVPA, despite the recommendation that 50% of time in PE should be MVPA [7, 46].

Preliminary findings of the outcome measures from the present study suggested that the intervention may have had a small to medium effect on physical activity levels, FMS inhibition and maths fluency. It should be noted that for all outcome measures, these findings are preliminary. This study was not sufficiently powered to demonstrate significant intervention effects but to help power a future definitive cluster RCT as noted above. The preliminary analyses were conducted largely to inform a future power calculation.

This present study follows on from a pragmatic evaluation of the ‘Go2Play Active Play’ intervention conducted by the authors of the present study [18]. Our previous study was a pragmatic evaluation (with a non-randomised small comparison group) of the intervention which lasted 5 months and involved two sessions per week [18]. The intervention tested in the present study used the same format (i.e. 30 min of facilitated games and 30 min of free play); however, the frequency of the sessions and the duration of the intervention were reduced to 10 weeks, one session per week so that it could be delivered to a larger number of schools. The previous evaluation of the active play intervention found a 16% increase in light intensity physical activity and a 3% increase in MVPA in the intervention group during an average school day [18]. However, in the present study, light intensity physical activity only increased by 0.7% in the intervention group and by 1.7% in the control group during an average school day. Percent time spent in MVPA increased by 1.4% (4.2 min) in the intervention group and by 0.6% (1.7 min) for the control group during an average school day. Given the low levels of MVPA in the intervention group at baseline (8.8% of the school day) in the present study, there should be scope to increase MVPA levels during school hours. The decision to reduce the intervention to 10 weeks was one made by the funders so that the intervention could be delivered to more schools; however, preliminary findings from the present study highlight that more sessions per week may be required to have a more meaningful effect on school day physical activity.

Physical activity is underpinned by competency in FMS as it has been suggested that children who have a higher competency in FMS are more likely to be physically active [22, 23]. Baseline FMS in the present study were poor, participants in the intervention group had a mean GMQ score of 87.7 and 92.0 in the control group; it is recommended that children should be scoring at least 100 [40]. The effect of the intervention on GMQ score (*d* = 0.4) was small, but the control group had no increase in their score, whereas the intervention group had an increase of 3.1 (95% CI 0.9, 5.3). These findings were similar to those reported by Adamo et al., who conducted a 6-month preschool intervention aimed at providing more active and outdoor play opportunities [20]. They found that the intervention group increased their GMQ score by 4.2 (95% CI 0.5, 7.9) and the control group had a small decrease of − 1.5 (95% CI − 4.82 to 1.77) [20]. The previous study conducted by Johnstone et al. found much larger increases in GMQ Score (+ 10.1; 95% CI 7.9, 12.3) for the intervention group and a small increase in the comparison group (+ 3.6–1.3 to 8.4), but the participants had lower baseline scores than the present study and the duration of the intervention was 5 months.

Recent research has suggested a possible link between MVPA and cognitively engaging activities (i.e. activities which target FMS) and improved executive function (inhibition) and attainment (particularly maths related outcomes) [24, 26]. The present study found preliminary evidence of a limited intervention effect on conflict scores (the measure of inhibition) for the fish trials (*d* = 0.1) or the arrow trials (*d* = 0.1). The present study found no intervention effect on children’s maths fluency scores. There was also a small intervention effect on addition (*d* = 0.3) and subtraction scores (*d* = 0.1). These scores are likely to have improved through daily maths lessons over the study period and there might potentially have been a practice effect.

There is some evidence of the impact of physical activity interventions on children’s inhibition and maths achievement. In the Medical College of Georgia randomised controlled trial, overweight (> 85th percentile) participants were recruited and assigned to either low-dose exercise (20 min/day), high-dose exercise (40 min/day) or a control condition (educational component). The exercises focused on fun, inclusion and intensity, and participants wore heart rate monitors to measure the intensity of physical activity. Executive functions (planning, attention, etc.) and maths attainment were assessed, and the authors found a dose-response relationship with planning (*p* = 0.015), but not maths achievement (*p* = 0.06). There were no significant intervention effects for other executive functions, but the authors did find significant effects on maths fluency (*p* = 0.01).

A study conducted by Donnelly et al. aimed to determine the effects of a 3-year physical activity intervention, consisting of 90 min of academic active lessons throughout the school week, on participants’ maths fluency using the WIAT II [47]. They found significant improvements in maths achievement in the intervention group compared to the control by approximately seven points [47]. Findings from the Georgia Trial and Donnelly et al. suggest that the present active play intervention requires a higher frequency of delivery per week and/or a longer duration to have a meaningful impact on cognition and academic attainment. However, the play intervention sessions in the present study were characterised by relatively high levels of MVPA and cognitively engaging activities, suggesting improvements in inhibition and maths fluency might be likely if more sessions were delivered per week. A future study may also benefit from utilising a more comprehensive method of assessing maths achievement, such as the WIAT II, rather than solely maths fluency; although, there are practical advantages of using a simpler measure. Furthermore, a future study should consider measuring social and emotional outcomes as these might be other important benefits of the intervention; these factors are thought to be important mediators for the relationship between physical activity and cognition [48]. Anecdotally, teachers have reported that the active play sessions improved friendships among children, improved happiness and general mental wellbeing.

### Study limitations

The present study was a feasibility cluster RCT aimed at informing a future definitive trial. Although this study had a high consent rate, low attrition, was well organised and had a strong design necessary for the development of a future definitive RCT [27], it had some important weaknesses.

Firstly, the lead researcher who collected most of the data could not be blinded to group allocation. It is unlikely that this impacted the physical activity, inhibition and maths fluency outcomes as the researcher could not influence these findings; however, there may have been a bias and/or human error for the FMS scoring. These were minimised by using a researcher with extensive experience in using the TGMD-2 and following standardised procedures. Future studies should either blind the researcher to the intervention and control groups, or film participants performing the FMS test and score using the recordings to improve the accuracy of the scores, which would improve intra- and inter-rater reliability. The school staff could also not be blinded to group allocation and, therefore, control schools may have compensated by encouraging more physical activity during break times or providing additional opportunities to enhance their math ability.

The schools which agreed to participate in the intervention had low SES, which may have limited the generalizability of the present study. The aim of the funder was to provide active play to the most deprived schools in Glasgow and for this reason, schools were eligible for this study if 70% or more pupils were from the 20% of Scotland’s most deprived areas. However, the majority of the schools who agreed to participate in the intervention were deprived and, therefore, schools did not differ with regards to deprivation. A future definitive RCT of this intervention might consider a wider cross-section of schools.

Finally, due to time and cost, the authors did not include additional outcomes to determine if there were any unintended intervention effects on participant’s social and emotional development. A future definitive trial should consider additional outcomes (including qualitative measures) to determine if there are any unintended intervention outcomes.

## Conclusions

The present feasibility study provided useful knowledge about the process and implementation of the intervention and trial procedures for the proposed definitive trial. The procedures for collecting the outcome data appear acceptable; however, further enhancements could be made for the FMS outcome measure to increase reliability and validity and to measure physical activity over a whole day to determine if there are intervention effects on physical activity outside of school hours. Most changes required for a definitive trial centre on the intervention itself; results suggest that a typical active play session generates a high amount of MVPA, but more than one session per week is needed to have a meaningful impact on the outcomes measured. Thought would need to be given on how to implement more active play sessions per week; it might be that the intervention is delivered twice per week over a 5–10-week period initially and then the intervention would then taper off during school hours and be offered as an after-school club. This provides a greater opportunity to offer more sessions per week and to have more influence over children’s physical activity levels outside of school hours. It might also be useful to incorporate a parental component; these two suggestions should be tested before a definitive trial takes place.

## Abbreviations

FMS: Fundamental movement skills
ISRCTN: The International Standardised Randomised Controlled Trials Number register
MVPA: Moderate-to-vigorous physical activity
RCT: Randomised controlled trial
SES: Socio-economic status
SIMD: Scottish index of multiple deprivation
TGMD-2: Test of Gross Motor Development-2

## References

[1] Department of Health (2011). StartActive, Stay Active.

[2] Tremblay MS, Carson V, Chaput JP, Connor Gorber S, Dinh T, Duggan M (2016). Canadian 24-hour movement guidelines for children and youth: an integration of physical activity, sedentary behaviour, and sleep. Appl Physiol Nutr Metab..

[3] Timmons BW, Leblanc AG, Carson V, Connor Gorber S, Dillman C, Janssen I (2012). Systematic review of physical activity and health in the early years (aged 0-4 years). Appl Physiol Nutr Metab.

[4] Janssen I, Leblanc AG (2010). Systematic review of the health benefits of physical activity and fitness in school-aged children and youth. Int J Behav Nutr Phys Act.

[5] Healthy Behaviours in School Children (HBSC). Findings from the HBSC 2014 survey in Scotland. 2015. http://www.cahru.org/content/03-publications/04-reports/hbsc_nr14_interactive_final.pdf. (accessed 21 June 2016).

[6] Tremblay MS, Barnes JD, González SA, Katzmarzyk PT, Onywera VO, Reilly JJ (2016). Global matrix 2.0: report card grades on the physical activity of children and youth comparing 38 countries. J Phys Act Health.

[7] Hollis JL, Williams AJ, Sutherland R, Campbell E, Nathan N, Wolfenden L (2016). A systematic review and meta-analysis of moderate-to-vigorous physical activity levels in elementary school physical education lessons. Prev Med.

[8] Reilly JJ, Johnston G, McIntosh S, Martin A (2016). Contribution of school recess to daily physical activity: systematic review and evidence appraisal. Health Behav Policy Rev.

[9] Martin A, Boyle J, Corlett F, Kelly P, Reilly JJ (2016). Contribution of walking to school to individual and population moderate-vigorous intensity physical activity: systematic review and meta-analysis. Pediatr Exerc Sci.

[10] Johnstone A, Hughes AR, Martin A, Reilly JJ (2018). Utilising active play interventions to promote physical activity and improve fundamental movement skills in children: a systematic review and meta-analysis. BMC Public Health.

[11] Janssen I (2014). Active play: an important physical activity strategy in the fight against childhood obesity. Can J Public Health.

[12] Truelove S, Vanderloo LM, Tucker P. Defining and measuring active play among young children: a systematic review. Journal of physical activity and health. 2017;14(2):155-66. 10.1123/jpah.2016-0195 27775475

[13] Gray C, Gibbons R, Larouche R, Sandseter EB, Bienenstock A, Brussoni M (2015). What is the relationship between outdoor time and physical activity, sedentary behaviour, and physical fitness in children? A systematic review. Int J Environ Res Public Health.

[14] Brussoni M, Gibbons R, Gray C, Ishikawa T, Sandseter EB, Bienenstock A (2015). What is the relationship between risky outdoor Play and health in children? A systematic review. Int J Environ Res Public Health.

[15] Cooper AR, Page AS, Wheeler BW, Hillsdon M, Griew P, Jago R (2010). Patterns of GPS measured time outdoors after school and objective physical activity in English children: the PEACH project. Int J Behav Nutr Phys Act.

[16] Scottish Health Survey. The Scottish Health Survey: 2016 Edition. Edinburgh: The Scottish Government; 2017. https://www.gov.scot/Resource/0052/00525472.pdf (accessed 21 June 2016)

[17] Veitch J, Bagley S, Ball K, Salmon J (2006). Where do children usually play? A qualitative study of parents’ perceptions of influences on children's active free-play. Health Place.

[18] Johnstone A, Hughes AR, Janssen X, Reilly JJ (2017). Pragmatic evaluation of the Go2Play active play intervention on physical activity and fundamental movement skills in children. Prev Med Rep.

[19] Goldfield GS, Harvey AL, Grattan KP, Temple V, Naylor PJ, Alberga AS (2016). Effects of child care intervention on physical activity and body composition. Am J Prev Med.

[20] Adamo KB, Rutherford JA, Goldfield GS (2010). Effects of interactive video game cycling on overweight and obese adolescent health. Appl Physiol Nutr Metab.

[21] Lubans DR, Morgan PJ, Cliff DP, Barnett LM, Okely AD (2010). Fundamental movement skills in children and adolescents: review of associated health benefits. Sports Med.

[22] Robinson LE, Stodden DF, Barnett LM, Lopes VP, Logan SW, Rodrigues LP (2015). Motor competence and its effect on positive developmental trajectories of health. Sports Med.

[23] Stodden D, Goodway J (2007). The dynamic association between motor skill development and physical activity. J Phys Educ, Recreat Dance.

[24] McMorris T, Tomporowski PD, Audiffren M (2009). Exercise and cognitive function.

[25] Pesce C, Masci I, Marchetti R, Vazou S, Sääkslahti A, Tomporowski PD (2016). Deliberate play and preparation jointly benefit motor and cognitive development: mediated and moderated effects. Front Psychol.

[26] Tomporowski PD, McCullick B, Pesce C. Enhancing children’s cognition with physical activity games. Champaign: Human Kinetics; 2015.

[27] Craig P, Dieppe P, Macintyre S, Michie S, Nazareth I, Petticrew M (2008). Developing and evaluating complex interventions: the new Medical Research Council guidance. BMJ..

[28] Eldridge SM, Chan CL, Campbell MJ, Bond CM, Hopewell S, Thabane L (2016). CONSORT 2010 statement: extension to randomised pilot and feasibility trials. Pilot Feasibility Stud.

[29] Reilly JJ, Kelly L, Montgomery C, Williamson A, Fisher A, McColl JH (2006). Physical activity to prevent obesity in young children: cluster randomised controlled trial. BMJ..

[30] The Scottish Government (2016). The Scottish Index of Multiple Deprivation.

[31] International Physical Literacy Association (2017). Retrieved from https://www.physical-literacy.org.uk/ (accessed 31 Jul 2016).

[32] Whitehead M (2001). The concept of physical literacy. Eur J Phys Educ.

[33] Whitehead M. Definition of physical literacy and clarification of related issues. ICSSPE Bulletin. 2013;65(1.2)

[34] The Aspen Institute (2015). Sport for all, Play for life: a playbook to get every kid in the game.

[35] Evenson KR, Catellier DJ, Gill K, Ondrak KS, McMurray RG (2008). Calibration of two objective measures of physical activity for children. J Sports Sci.

[36] Sasaki JE, John D, Freedson PS (2011). Validation and comparison of ActiGraph activity monitors. J Sci Med Sport.

[37] Hänggi JM, Phillips LR, Rowlands AV (2013). Validation of the GT3X ActiGraph in children and comparison with the GT1M ActiGraph. J Sci Med Sport.

[38] Trost SG, Loprinzi PD, Moore R, Pfeiffer KA (2011). Comparison of accelerometer cut points for predicting activity intensity in youth. Med Sci Sports Exerc.

[39] Kwon S, Mason M, Welch S (2015). Physical activity of fifth to sixth graders during school hours according to school race/ethnicity: suburban Cook County, Illinois. J Sch Health.

[40] Ulrich DA (2000). Test of gross motor development-2.

[41] Weintraub S, Dikmen SS, Heaton RK, Tulsky DS, Zelazo PD, Bauer PJ (2013). Cognition assessment using the NIH toolbox. Neurology..

[42] Westwood F. Drilling basic number facts: should we or should we not? Aust J Learn Diffic. 2003;8:12-8.

[43] O'Dwyer MV, Fairclough SJ, Knowles Z, Stratton G (2012). Effect of a family focused active play intervention on sedentary time and physical activity in preschool children. Int J Behav Nutr Phys Act.

[44] Engelen L, Bundy AC, Naughton G, Simpson JM, Bauman A, Ragen J (2013). Increasing physical activity in young primary school children—it's child's play: a cluster randomised controlled trial. Prev Med.

[45] Brazendale K, Chandler JL, Beets MW, Weaver RG, Beighle A, Huberty JL (2015). Maximizing children's physical activity using the LET US Play principles. Prev Med.

[46] Association for Physical Education (2008). http://www.afpe.org.uk/physical-education/wp-content/uploads/afPE_Health_Position_Paper_Web_Version2015.pdf (accessed 17 June 2016).

[47] Donnelly JE, Greene JL, Gibson CA, Smith BK, Washburn RA, Sullivan DK (2009). Physical activity across the curriculum (PAAC): a randomized controlled trial to promote physical activity and diminish overweight and obesity in elementary school children. Prev Med.

[48] Diamond A (2013). Executive functions. Annu Rev Psychol.

